# Trends in Pediatric Surgery in the New Millennium

**Published:** 2020-05-31

**Authors:** A Garzi, M. S Rubino

**Affiliations:** Division of Pediatric M.I.S. and Robotic Surgery University of Salerno, Italy

Over the past 35 years, there have been extraordinary progresses in various areas of surgery. It is a matter of fact that conservative surgery in the treatment of breast cancer, minimally invasive laparoscopic or thoracoscopic surgery, organ transplantation, will become a part of the history of surgery.

In major pediatric surgery, international literature showed a significant reduction in mortality and morbidity after the surgical correction of the most complex congenital malformations. At the beginning of the 1980s, there were significant complications and high mortality rates even in the reference centres for these diseases, whereas today, in the same centres, mortality is less than 2% and there is a clear reduction in the incidence of post-operative complications.

A necessary condition for obtaining these results is the experience and the specific competence of the surgical team, in addition to the environment and the presence of other skilled specialists to consult.

It is easy to understand why this is the result of a long and intense technological and clinical research. In fact, in the recent past, the physiopathological knowledge of the diseases, the diagnostic investigations, the methods of preparing the patient for the surgery, the anesthesiological and resuscitation techniques and the postoperative treatments have been improved.

At the same time, new surgical instruments were developed and, therefore, have been adopted such as surgical stapler, Argon plasma coagulation, ultrasonic dissectors and scalpels, radiofrequency instruments, ultrasound and intraoperative endoscopes. These technological innovations, in addition to the safety of surgery, lead to a considerable reduction in the surgical time and in the anaesthesia time.

Improvement has also been made in pediatric surgery of medium gravity: laparoscopic surgery and other minimally invasive techniques are certainly the most revolutionary novelties of the recent years. The laparoscopic appendicectomy and cholecystectomy, nowadays, have almost replaced the traditional laparotomic surgery.

Although with some limitations, the experience over the past 30 years, has shown that laparoscopic technique can be extended to the treatment of several diseases of lungs, oesophagus, colon, adrenal glands, spleen, kidney and other peritoneal and retroperitoneal disorders.

Generally, the course indicated by minimally invasive surgery is certainly irreversible because of its high utility in many aspects. However, we believe that there is still the need to improve the results achieved with these techniques.

Minimally invasive surgery is less damaging and allows a short-term hospitalization and a reduction of recovery time. Moreover, in order to increase the results, we suggest to apply corrective measures to the current procedures like an internal audit and a periodic review of the results of each individual surgeon, training methods which ensure a short learning curve, and adaptation of hospital centres in order to exploit the technological innovation.

In this special issue the aim is to focus on the minimally invasive pediatric surgery program performed at the University Hospital of Salerno, in collaboration with the most prestigious National centres, in order to create a highly qualified network.

Furthermore, the aim of the program is to improve quality of care for patient, to prevent from extraregional care migration and to offer high-level training opportunities thanks to the circulation of surgeons and teachers. As a results, also economic asset would be contained.

Consequently, it is highly predictable that, as the new techniques are currently proposed at high speed, sometimes it does not allow to measure the effectiveness or to predict the effects of the new procedures, as it could happen for the introduction of robotics in pediatric surgery. Therefore, the desire to evaluate a technology at an early stage of its development necessarily implies making decisions on an initially incomplete knowledge base.

However, we believe that technological applications should be guided by common sense and guaranteed by careful training.

In conclusion, during the training, a surgeon unavoidably needs to learn also traditional techniques, because the possible complications of the new surgical techniques may be corrected almost exclusively through the adoption of traditional open-techniques and, above all, by the experience of the surgeon.

